# ANTICIPATED NEED FOR NURSING HOME PLACEMENT AMONG LESBIAN, GAY, BISEXUAL ADULTS AGES 50-64: FINDINGS FROM THE HRS

**DOI:** 10.1093/geroni/igz038.568

**Published:** 2019-11-08

**Authors:** Mekiayla Singleton, Zach Gassoumis, Susan Enguidanos

**Affiliations:** 1 Leonard Davis School of Gerontology, University of Southern California, Los Angeles, California, United States; 2 University of Southern California, Los Angeles, California, United States

## Abstract

By 2030, the population of LGBTQ older adults is expected to exceed 6 million. Yet little is known about the expected use of nursing homes (NH) among LGBTQ older adults. Prior research has found NHs lack cultural sensitivity, and that LGBTQ NH residents are going “back into the closet” and not disclosing their sexual orientation due to discrimination and quality of care concerns. Using data from 2016 HRS, we describe bivariate differences between the LGB and heterosexual population, ages 50 to 64, and conduct a linear regression to determine the impact of LGB status on self-reported chance of moving to a NH in the future. Compared with the heterosexual population (n=4,049), these LGB adults (n=158) had a higher mean self-reported chance of moving to a NH (p<.01), fewer children (p<.01) and reported a slightly higher health rating (p<.05). LGB adults ages 50-64 also were more likely to be unmarried (71%, p< .001), white (59%, p< .001) and have a college degree (51%, p<.001). After controlling for sociodemographic variables, there were no significant differences between LGB and heterosexual adults’ self-reported chance of moving to a NH. Although anticipated chance of moving to a NH is no different for LGB adults ages 50-64 when controlling for their sociodemographic profiles, as a group they have a higher anticipated chance than heterosexual adults. These findings support the need for improved education, training, and structural changes within long-term care settings to better serve the growing older adult LGB population.

